# A novel multi‐modality imaging phantom for validating interstitial needle guidance for high dose rate gynecological brachytherapy

**DOI:** 10.1002/acm2.14075

**Published:** 2023-06-19

**Authors:** Brett Eckroate, Diandra Ayala‐Peacock, Rajesh Venkataraman, Sabrina Campelo, Junzo Chino, Sarah Jo Stephens, Yongbok Kim, Sheridan Meltsner, Julie Raffi, Oana Craciunescu

**Affiliations:** ^1^ Department of Radiation Oncology Rutgers University New Brunswick New Jersey USA; ^2^ Department of Radiation Oncology Duke University Medical Center Durham North Carolina USA; ^3^ Eigen Health Services, LLC Grass Valley California USA; ^4^ Virginia Tech‐Wake Forest School of Biomedical Engineering and Sciences Blacksburg Virginia USA

**Keywords:** 3D printing, 3D ultrasound, computed tomography, gynecological brachytherapy, magnetic resonance, pelvic phantom

## Abstract

**Purpose:**

To design, manufacture, and validate a female pelvic phantom for multi‐modality imaging (CT, MRI, US) to benchmark a commercial needle tracking system with application in HDR gynecological (GYN) interstitial procedures.

**Materials and Methods:**

A GYN needle‐tracking phantom was designed using CAD software to model an average uterus from a previous patient study, a vaginal canal from speculum dimensions, and a rectum to accommodate a transrectal ultrasound (TRUS) probe. A target volume (CTV_HR_) was designed as an extension from the cervix‐uterus complex. Negative space molds were created from modeled anatomy and 3D printed. Silicone was used to cast the anatomy molds. A 3D printed box was constructed to house the manufactured anatomy for structural integrity and to accommodate the insertion of a speculum, tandem, needles, and TRUS probe. The phantom was CT‐imaged to identify potential imperfections that might impact US visualization. Free‐hand TRUS was used to guide interstitial needles into the phantom. The commercial tracking system was used to generate a 3D US volume. After insertion, the phantom was imaged with CT and MR and the uterus and CTV_HR_ dimensions were verified against the CAD model.

**Results/Conclusions:**

The manufactured phantom allows for accurate visualization with multiple imaging modalities and is conducive to applicator and needle insertion. The phantom dimensions from the CAD model were verified with those from each imaging modality. The phantom is low cost and can be reproducibly manufactured with the 3D printing and molding processes. Our initial experiments demonstrate the ability to integrate the phantom with a commercial tracking system for future needle tracking validation studies.

## 1 INTRODUCTION

The current gold standard for modern hybrid intracavitary and interstitial HDR procedures for gynecological malignancies includes imaging with MRI with or without CT for treatment planning.^1^, ^2^, ^3^, ^4^, ^5^ When free‐hand applicator and needle insertion techniques are used, it is an iterative process, where the implant quality is evaluated with post‐implant imaging. Adjustments or re‐insertion may be considered if the initial implant is sub‐optimal.

Several real‐time image guidance techniques for applicator implantation have been implemented or proposed including ultrasound, fluoroscopy, and electromagnetic tracking.^1^, ^5^, ^6^, ^7^, ^8^, ^9^, ^10^ Fluoroscopy is a form of ionizing radiation, but it provides good depth visibility for fiducial‐based targets. Ultrasound and electromagnetic tracking, as non‐ionizing imaging modalities, are attractive modalities for image‐guided brachytherapy. Ultrasound has good soft tissue contrast, but is limited in its depth visualization. Van Dyk et al. reviewed transrectal ultrasound (TRUS) and transabdominal ultrasound (TAUS) use in gynecological (GYN) brachytherapy showing that ultrasound images can be used to improve technical quality of implants.^1^ Sharma et al. showed in a patient study that TRUS is an effective tool for image‐guided HDR.^11^

While 2D TRUS is a feasible tool for real‐time image guidance,^1^, ^11^, ^12^ there are commercial 3D ultrasound steppers that can also aid in needle tracking and guidance^5^, ^13^ and eliminate the need for free‐hand US probe movement. Precise rotation and translation are available using 3D TRUS and can aid needle insertion accuracy. Such technology have been used in other disease sites, for example, Artemis robotic system developed by Eigen Health Services (Grass Valley, CA) integrates 2D TRUS probe with mechanical tracking system for prostate biopsies.^14^ Artemis has been shown to be successful in generating 3D US volumes of the prostate and localizing needles to track and guide them to targeted biopsy sites.^14^, ^15^, ^16^

Currently, there are no commercial female pelvic medical phantoms that allow both multi‐modality imaging and HDR simulation. Some in‐house female pelvic phantom studies have been published for HDR brachytherapy due to a lack of commercial phantoms available.^17^ Nattagh et al. built a realistic pelvic phantom using rubber and a gelatin matrix to mimic soft tissue.^18^ The purpose of this phantom was to aid in resident comfort for ultrasound‐guided needle placement and suturing. Rodgers et al. developed a pelvic phantom to test the feasibility of 3D TRUS for gynecological malignancies.^5^ The pelvic phantom was created using agarose gelatin for the uterus and rigid structures for the vaginal canal and rectum. 3D TRUS was validated by calculating inserted needle trajectories and comparing those to CT.

Our previous work^19^, ^20^ utilized 3D printing techniques to create anatomically accurate training phantoms for medical residents to practice applicator insertions for HDR GYN brachytherapy. For this study, the patient anatomy and manufacturing techniques developed previously were adapted to prototype a novel multi‐modality imaging phantom that will aid in simulating intracavitary/interstitial GYN HDR procedures utilizing real‐time TRUS guidance. The GYN needle‐tracking phantom incorporates all relevant anatomy: vaginal canal, cervix, uterus, and rectum to allow for applicator, needle, and US probe insertion and is compatible with multi‐modality imaging including CT, MRI, and US. A small high risk clinical target volume (CTV_HR_) was also incorporated to provide a target for needle insertion. The target was created to represent gross parametrial disease beyond the cervix, which in addition to the cervix is defined as CTV_HR_.

## 2 MATERIALS AND METHODS

### 2.1 Design goals

We had the following design goals for the GYN needle‐tracking phantom: (1) include relevant anatomy for GYN HDR procedures, (2) use pliable, tissue‐mimicking materials that allow realistic insertion of applicators and TRUS probe, (3) allow reproducible and low‐cost production, (4) achieve good image quality with CT, MR, and US imaging modalities. In order to achieve the design goals, 3D printing and silicone casting techniques were used to model the female pelvic anatomy.

### 2.2 Female pelvic structures: uterus, vaginal canal, rectum, and CTVHR

The GYN needle‐tracking phantom incorporated an average uterus based on a previous patient data study conducted by our group.^19^ The study included taking dimensional measurements of uteri from 50 patient MRI and CT scans. The average uterus dimensions are shown in Table 1. The vaginal canal designed for the GYN needle‐tracking phantom was based on the dimensions of a KleenSpec® 590 Series Vaginal Speculum (Welch Allyn, Skaneateles, NY) to allow for applicator and needle insertion. The vaginal canal proximal height was 5.74 cm and the distal height was 7.34 cm. The overall length of the vaginal canal was 6.81 cm and the width was 2.94 cm. The designed rectum was based on a BK Medical Endocavity Biplane 8848 US Probe (BK Medical Holding Company, Inc., Burlington, MA), with an inner diameter of 2 cm to allow for US probe insertion. Lastly, a CTV_HR_ was created as an extrauterine (parametrial) extension on the posterior lateral side. The CTV_HR_ for this phantom study was a small half‐ellipsoid structure with a volume of approximately 3 cm^3^. It is much smaller than the CTV_HR_ volumes observed in the patient study and is not meant to mimic typical disease presentation or adhere to the CTV_HR_ definition, which by definition includes the cervix. Instead, our phantom CTV_HR_ was designed as a separate structure for imaging and needle targeting purposes.

**TABLE 1:** Average uterus dimensions.^19^

Body length	7.87 cm
Body width (Proximal Cervix)	2.74 cm
Body width (Midpoint)	4.45 cm

### 2.3 3D modeling

Molding is a cost effective and easily reproducible technique that can incorporate materials with similar mechanical properties to pelvic anatomy, allowing the phantom to be clinically relevant.^21^ Autodesk Fusion 360 (Autodesk Inc, San Francisco, CA) was used to model the female pelvic anatomy. The computer aided design (CAD) files for the anatomy were Boolean subtracted from modeled solid bodies to create negative space molds. Once the CAD files were finalized, they were exported as stereolithography (STL) files to an Ultimaker S3 3D printer (Ultimaker BV, Netherlands).

### 2.4 Pelvic tissue materials

To simulate needle insertion into patient tissue, a tissue‐mimicking silicone casting material was used for the pelvic anatomy. Smooth‐On Mold Star™ Silicone 20T (Smooth‐On, Easton, PA), with an equivalent 20A shore value, has a Young's Modulus (0.32 MPa) similar to that of a human uterus (0.2–1.4 MPa). The silicone pelvic anatomy was surrounded by a background medium to allow ultrasound propagation. To minimize reflection and increase transmission of the ultrasound waves, an agar formulation with a similar acoustic impedance to silicone (1.03–1.27 Mrayl) was selected as a background medium.^14^ For ultrasound to propagate efficiently, the differences in acoustic impedance between materials should be minimized to avoid reflection. Adding cellulose to the agar increased the density of the background medium, thus raising the modulus and acoustic impedance. Glycerol was also added to the agar formulation to act as a preservative.

### 2.5 Construction of the GYN needle‐tracking phantom

To construct the GYN needle‐tracking phantom, silicone was used to cast the negative space molds. Smooth‐On Mold Star™ Silicone 20T comes in two parts, a silicone elastomer and a crosslinker. Equal parts of each component were mixed to cast the anatomy. Once mixed, the silicone had a cure time of 30 min.

The agar formulation was prepared by bringing 2.2 L of water to a boil. Once boiling, 70 g of agar powder, 160 cm^3^ of glycerol, and 30 g of cellulose were added to the water and stirred until dissolved. Once the agar formulation was dissolved and uniformly mixed, it was removed from heat and allowed to cool before using. In addition to serving as the background medium surrounding the anatomy, the agar was also used to create the CTV_HR_ and to fill the interior of the uterus. The uterus was designed as a hollow shell, with the external shell composed of silicone and filled with agar to allow for better US propagation and applicator insertion. Prior to filling the uterus, a CTV_HR_ was created by using a separate mold with a negative space extension from the uterus. Agar was poured into the mold to form the body of the CTV_HR_. After the agar hardened, silicone was used as an adhesive to seal the agar in place to the uterus due to the lack of adhesion between silicone and agar.

Once the uterus, CTV_HR_, and pelvic attachment were completed, they were placed back into the mold to secure their location and connected to the rectum and vaginal canal mold. The connection was maintained using registration pins, which allowed each mold to snap into its designed location. The individual mold pieces then were bound together with duct tape to minimize silicone leakage through the mold junction gaps. Once secured, the rectum and vaginal canal were cast. The uterus was attached prior to casting the rectum and vaginal canal to allow the cervix to be joined at the distal end of the vaginal canal at the desired location. The distal portions of the vaginal canal and rectum were cast together with a connecting pelvic attachment. Once the anatomy casting was complete, it was housed in a 3D printed box with openings to allow the anatomy to be inserted and the pelvic attachment to catch and maintain the positioning of the anatomy. Silicone was used to seal the pelvic attachment to the outside front face of the box and the rectum extending through the back face of the box.

After positioning and sealing the anatomy in place, the liquid agar formulation was added to fill the box surrounding the anatomy to provide structural integrity to the model and allowed for US propagation. The agar formulation solidified after cooling and the completed phantom was stored in a refrigerator to maintain the agar consistency and extend its useful life.

### 2.6 Multi‐modality imaging of needle‐tracking phantom

Once constructed, the GYN needle‐tracking phantom was imaged using a Siemens Biograph mCT (Siemens Healthcare, Erlangen, Germany) with a slice thickness of 0.6 mm. The CT image was used to inspect the phantom for irregularities like air pockets in the silicone, collapsed anatomy, and heterogeneities in the agar background, as well as verify that the dimensions of the anatomy matched the CAD model. After the initial CT scan, the phantom was imaged using a BK Flex Focus US system with a BK 8848 endocavity biplane US probe (BK Medical Holding Company, Inc., Burlington, MA) at 6 MHz for better depth visualization. Two needles were inserted under TRUS guidance by manually rotating and translating the US probe. One needle was inserted into the CTV_HR_, while the other was inserted into the agar‐filled uterus. After insertion, a tracking system developed by Eigen Health Services LLC was used to generate a 3D US volume of the uterus. The system was manually controlled to generate the volume by completing a 180° sweep of the pelvic anatomy. Similar to our clinical workflow, CT and MR images of the implanted needles were acquired following our clinical HDR GYN imaging protocols. The CT images were obtained to digitize the inserted needles and the MR images were used for target and normal tissue delineation. The MR images were acquired with a Siemens Skyra 3T MRI scanner (Siemens Healthcare, Erlangen, Germany) following our clinical HDR GYN imaging protocol using the body coil: T1w – 3d SPGR 1 mm isotropic (TR‐3.7, TE = 1.3), FOV 300 × 300, and T2w—axial (TR = 2000, TE = 121), FOV 300 × 300.

## 3 RESULTS

### 3.1 3D phantom modeling and completed phantom

**FIGURE 1:**
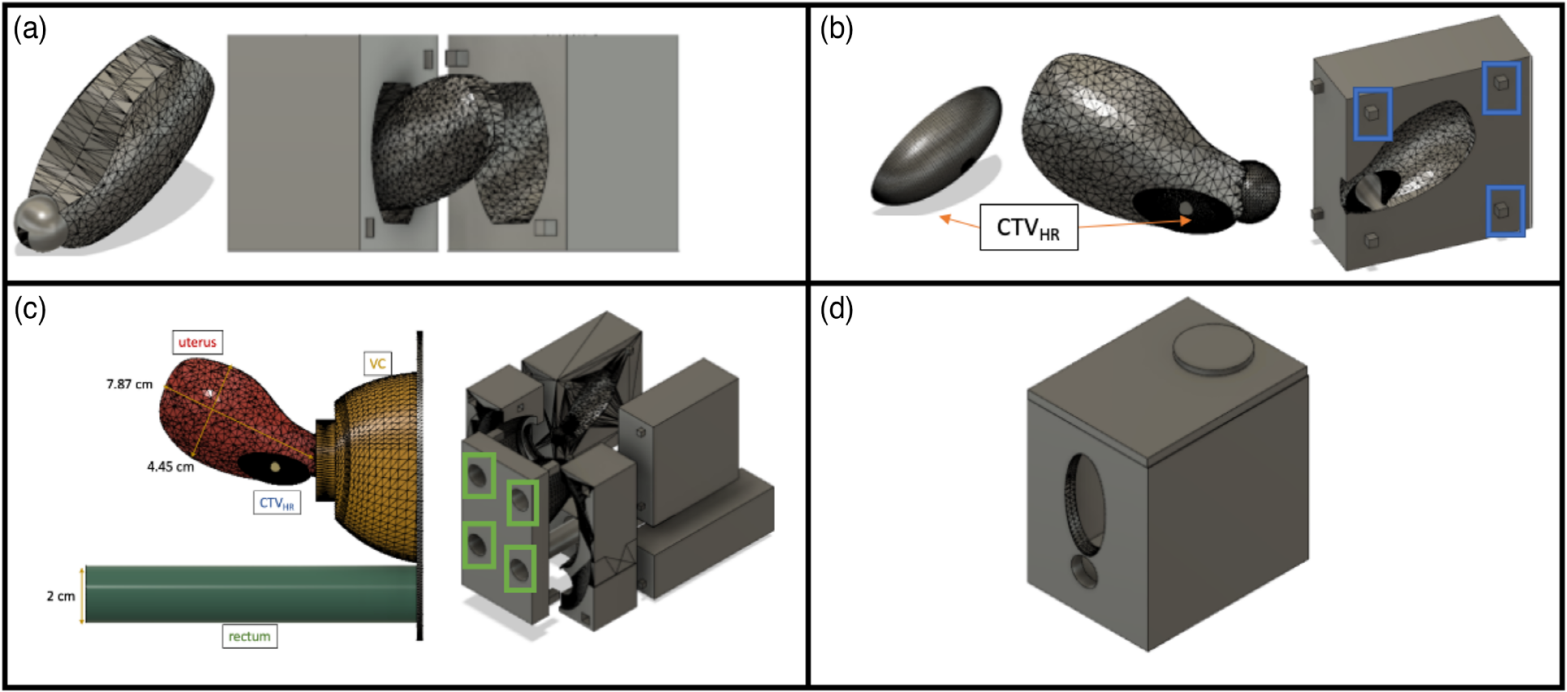
Autodesk Fusion360 CAD anatomy and molds: (a) uterus; (b) CTV_HR_ and uterus (the blue boxes represent registration pins that hold the mold together); (c) vaginal canal (VC), uterus and rectum (the green boxes represent holes to pour silicone in the mold for casting); (d) housing box.

**FIGURE 2:**
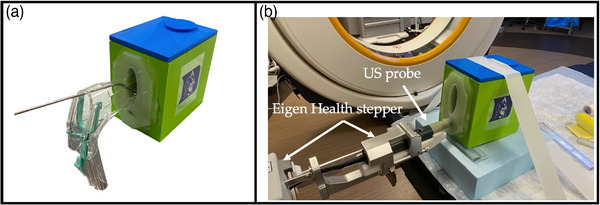
(a) Constructed GYN needle‐tracking phantom with a speculum and central tandem inserted; (b) 3D US setup with stepper developed by Eigen Health Services LLC, showing the robotic arm mounted to the couch with the endorectal 8848 BK US probe.

Figure 1 shows the pelvic anatomy and the respective molds: (a) uterus; (b) CTV_HR_ and uterus; (c) vaginal canal (VC), uterus and rectum, and (d) housing box as designed using Fusion 360 (Autodesk Inc, San Francisco, CA). The CAD dimensions of the uterus (Figure 1c) are 7.87 cm × 4.45 cm (length × width) with a volume of 76.8 cm^3^. The dimensions of the designed half ellipsoid CTV_HR_ (Figure 1b) are 1.50 cm × 3.00 cm × 1.25 cm (w × l × h) with a volume of 3.00 cm^3^. The completed phantom is shown in Figure 2a.

Constructing the entire phantom from printing the molds to casting and combining the anatomy can take roughly 3−4 days. Printing the molds can take anywhere from 2 to 20 h depending on complexity and size. Creating the silicone anatomy is done on a part‐by‐part basis where each anatomical part takes roughly 45 min to prepare the silicone and cast the mold.

### 3.2 Phantom evaluation on CT and US images

**FIGURE 3:**
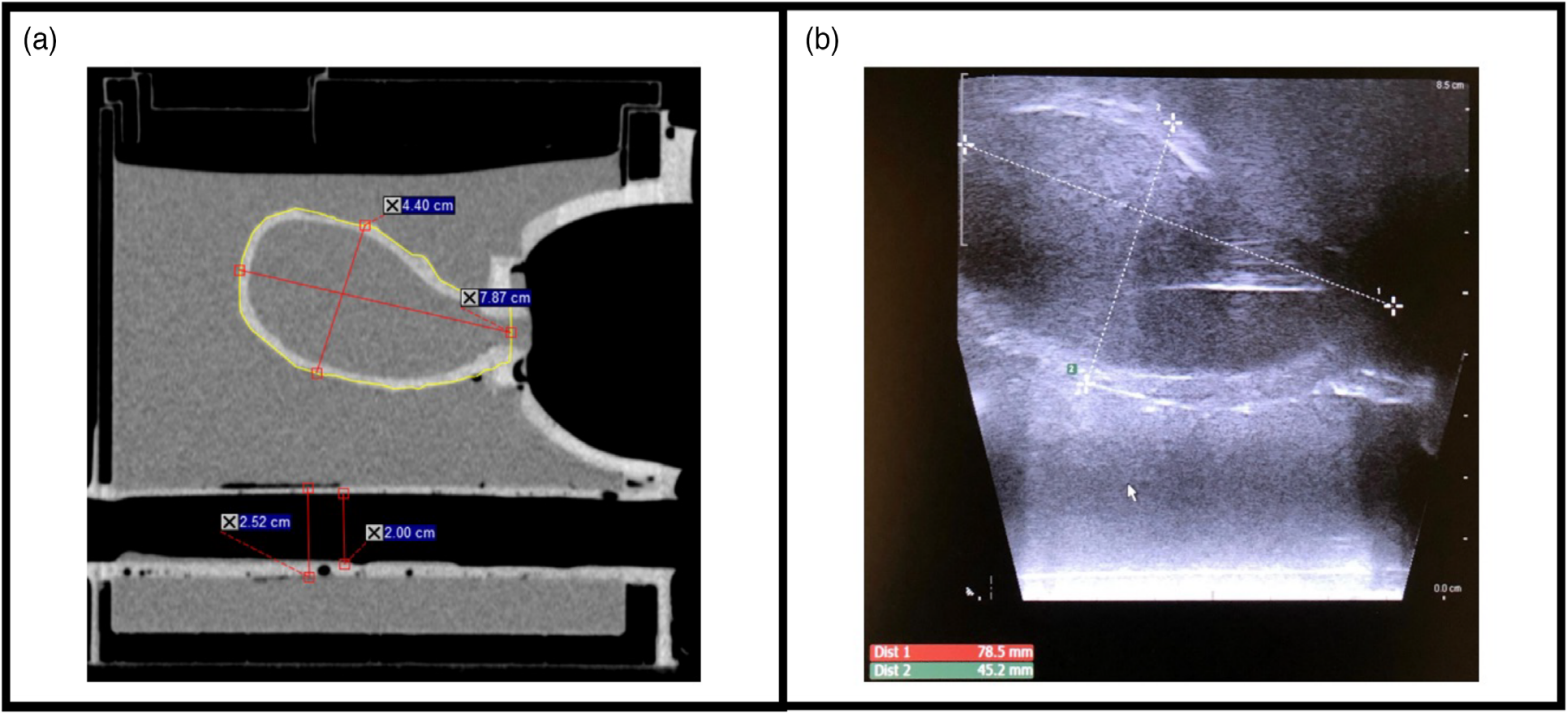
(a) Sagittal CT image showing measured uterus (length x width) and rectum; (b) Sagittal US image with uterus dimensions.

As shown in Figure 3a, the dimensions of the constructed uterus at its midpoint were 4.40 cm in width and 7.87 cm in length when measured on the CT images. The volume of the uterus as contoured on the CT images was 77.1 cm^3^. The inner diameter of the rectum measured 2.00 cm, while the outer diameter was 2.52 cm.

The phantom setup for the 3D US is shown in Figure 2b, where the stepper was mounted to the end of the procedure table. The BK 8848 TRUS probe was placed on the stepper and inserted into the phantom. Using the software associated with the stepper, a 3D ultrasound volume of the uterus was acquired by manually rotating the probe. After the acquisition, an approximate uterus was contoured on the 3D US dataset. Due to the limited penetration range of US, an approximate contour was drawn for the anterior boundary of the uterus. The uterus dimensions measured on a sagittal US image (Figure 3b) were 4.52 cm in width and 7.85 cm in length.

**FIGURE 4:**
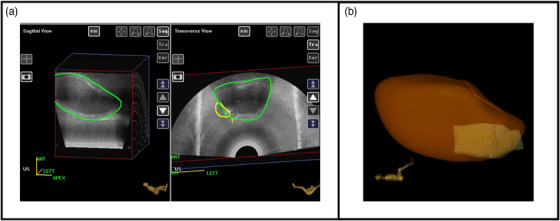
(a) US contour of the uterus (green) from the tracking software developed by Eigen Health Services LLC displayed in sagittal and axial planes. The axial plane also depicts the CTV_HR_ (yellow); (b) 3D‐rendered uterus (orange) and CTV_HR_ (yellow).

T2‐weighted MR images were fused with the US 3D image and the CTV_HR_ was transferred to the US image. Figure 4 shows the generated uterus (green) and CTV_HR_ (yellow) contours in the axial and sagittal planes, along with a 3D rendering of the two structures. The volume of the uterus as contoured on the US images was 62.2 cm^3^.

### 3.3 Phantom evaluation on CT and MR images

**FIGURE 5:**
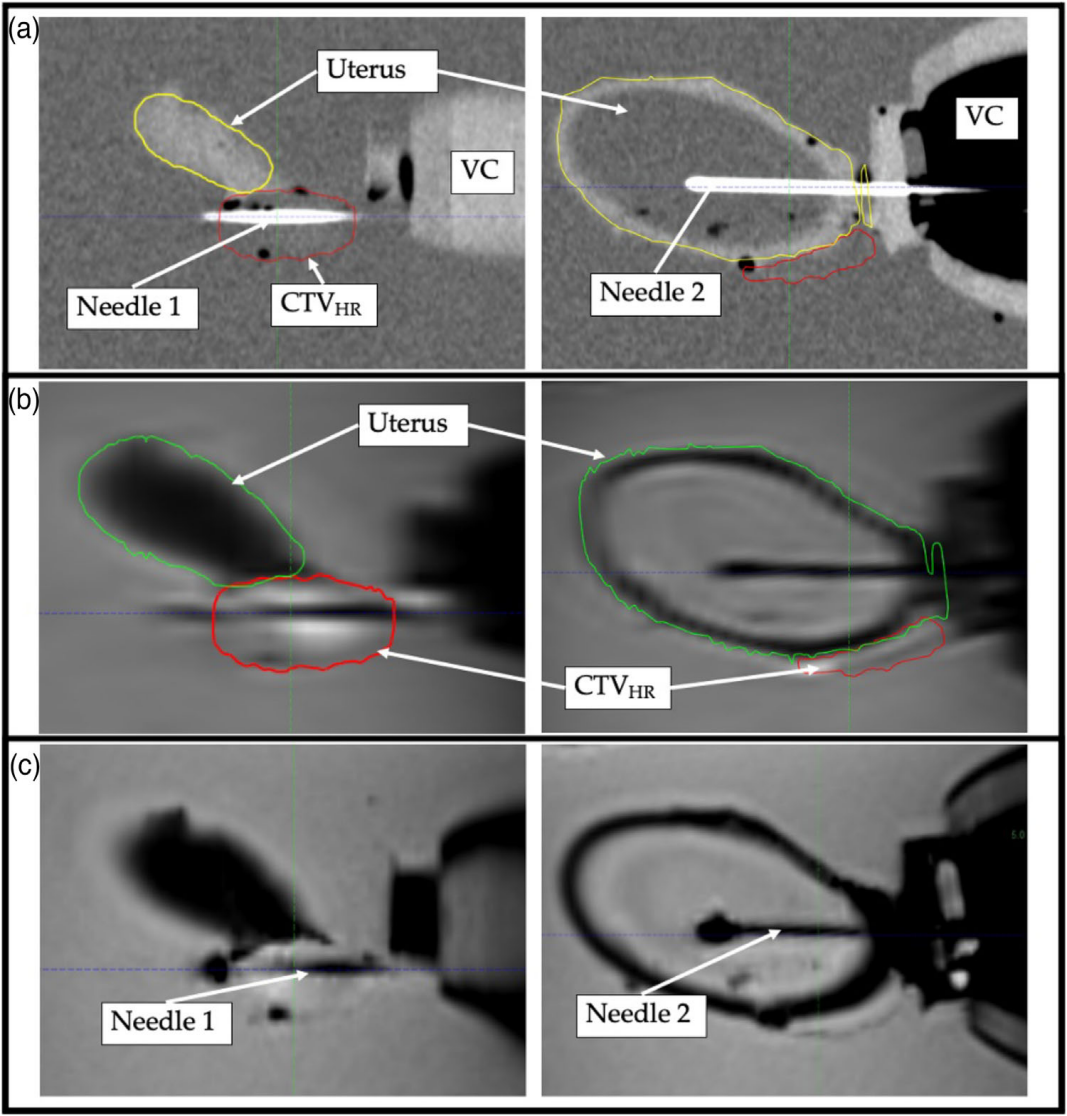
Two sagittal planes showing the pelvic anatomy and inserted needles: (a) CT; (b) T2‐weighted MR; (c) T1‐weighted MRI. VC represents the vaginal canal.

Figure 5 shows the CT, T1‐ and T2‐weighted sagittal images of the uterus and CTV_HR_, respectively. The CT images show the contoured anatomy and needles, the T1‐weighted MR image shows the two inserted needles, and the T2‐weighted MR image shows the contoured uterus and CTV_HR_. The volumes of the uterus and CTV_HR_ as contoured on the T2‐weighted image were 82.9 cm^3^ and 3.2 cm^3^, respectively.

Table 2 summarizes the uterus and CTV_HR_ dimensions from the CAD model and the completed phantom, as measured with the multiple imaging modalities. Note that the CTV_HR_ dimensions were only measured on the T2w MRI as to match the clinical workflow.

**TABLE 2:** Uterus and CTV_HR_ ^a^ dimensions.

	CAD	CT	US	T2W MRI
**Uterus length (cm)**	7.87	7.87	7.85	8.06
**Uterus width (cm)**	4.45	4.40	4.52	4.47
**Uterus volume (cm^3^)**	76.8	77.1	62.2^b^	82.9
**CTV_HR_ length (cm)**	3.0	NA	NA	2.56
**CTV_HR_ width (cm)**	1.5	NA	NA	1.48
**CTV_HR_ height (cm)**	1.25	NA	NA	1.15
**CTV_HR_ volume (cm^3^)**	3.0*	NA	NA	3.2

^a^ CTV_HR_ Dimensions only measured from T2w MRI as in clinical practice.

^b^ Approximate values—US volume was limited by incomplete visualization of the uterus and CTV_HR_ construction process led to slight deviation from modeled dimensions.

### 3.4 Phantom cost

The phantom is mold‐based and easily reproducible through 3D printing techniques, making it a cost effective tool. The overall cost for the raw phantom materials is roughly $140.00 (as of November 2022), which includes polylactic acid (PLA) for 3D printing, silicone, agar, cellulose, glycerol, and distilled water. This estimate does not include the price of a 3D printer, as we had access to a full‐service 3D printing laboratory at our institution. However, once printed, with the exception of the uterus, the 3D printed molds are reusable. The inner portion of the uterus mold was broken to allow removal and achieve the hollow design. Once constructed, however, the silicone uterus can be reused and refilled with agar as needed.

## 4 DISCUSSION

A multi‐modality GYN needle‐tracking phantom was designed and manufactured to demonstrate the feasibility of using transrectal ultrasound‐guided needle insertion for complex interstitial/intracavitary gynecological HDR procedures. In comparison to existing pelvic phantoms,^5^, ^18^ the GYN needle‐tracking phantom consists of relevant anatomy modeled from a patient data study,^19^ tissue‐mimicking materials for realistic applicator and endorectal probe insertion, and low‐cost materials. The designed phantom incorporates a representative female pelvic anatomy and consists of tissue‐mimicking materials for applicator insertion and interstitial needle implantation for simulation of HDR GYN procedures. Lastly, the phantom can be imaged with US, CT, and MR and can be integrated with a commercial 3D US system to implement needle tracking and guidance techniques. The needle tracking phantom, when compared to similar phantoms, is novel in that it includes tissue‐relevant materials for all pelvic anatomy and a target volume for needle tracking. The phantom anatomy was also modeled from a patient data study to include accurate structures to closely simulate an HDR procedure.

Several incremental design changes were made to the GYN needle‐tracking phantom to optimize US visibility. Gelatin and simple agar (without cellulose and glycerol) were tested as background media, and were found to provide little structural integrity and inferior US visualization. Adding cellulose made the agar stiffer which provided better structural support for the anatomy and more closely matched the acoustic impedance of the silicone for improved US visualization. We found that adding glycerol and refrigerating the final agar formulation extended the useful life of the agar, in terms of maintaining its homogeneous consistency and inhibiting bacterial growth. The agar will still degrade over time, but can be stored for at least a week after initial preparation. Prior to the final hollow design, the uterus was made entirely of silicone with a small uterine canal for tandem insertion. The thick silicone resulted in high US attenuation and prevented full visualization of the uterus. Finally, the rectal diameter was originally larger than the needed 2 cm for the US probe. The larger diameter allowed for ease of insertion and better range of motion, but resulted in poor contact between the probe and rectum. The diameter was decreased to maintain better contact.

**FIGURE 6:**
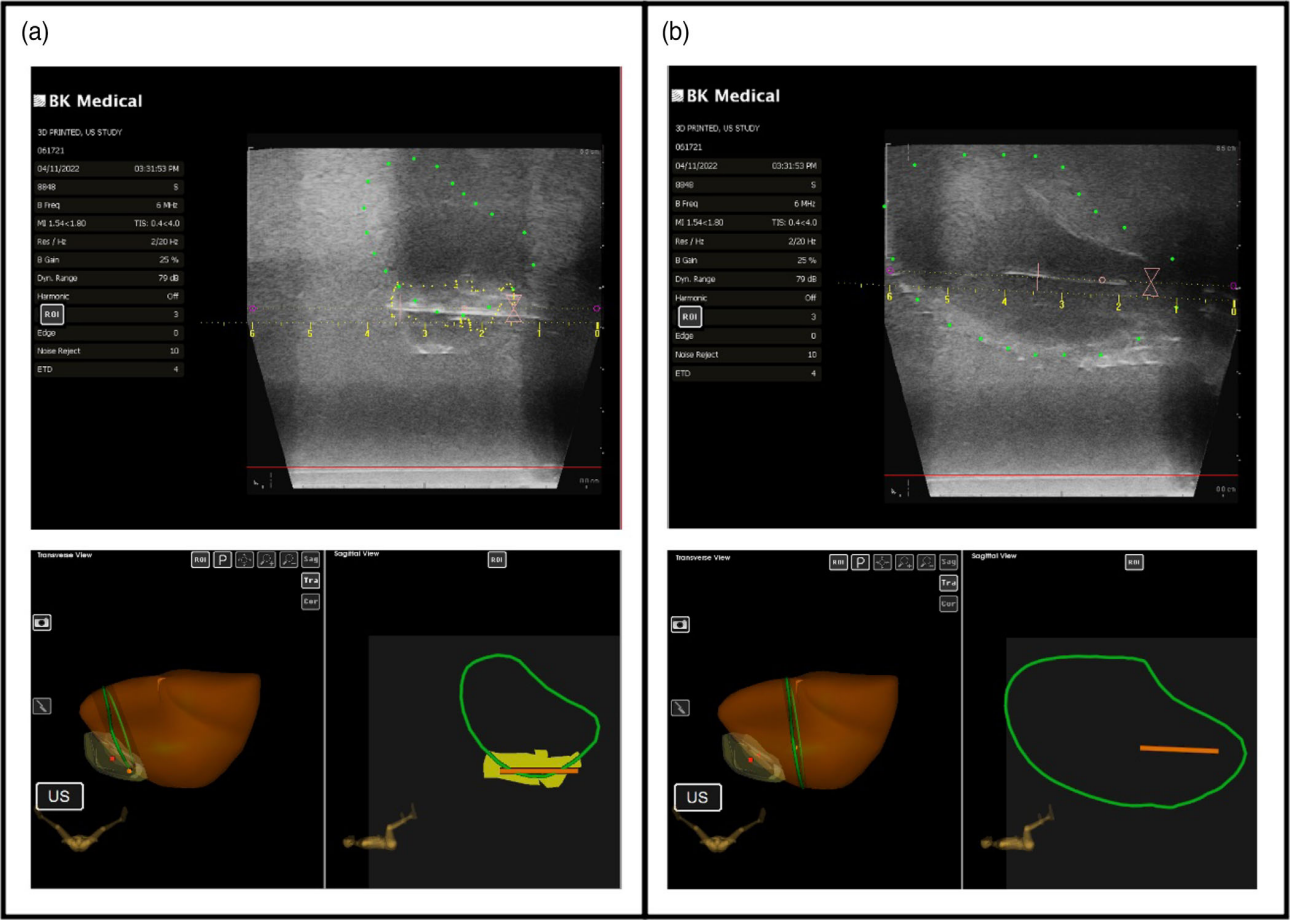
Sagittal US images and 3D and 2D renderings of the uterus, CTV_HR,_ and tracked needles: (a) needle inserted into CTV_HR_; (b) needle inserted into uterus.

The feasibility of using Eigen Health Services LLC's needle tracking software was also briefly tested with this first phantom prototype. Figure 6 shows the titanium needles as visualized on US, together with 3D renderings of the uterus, CTV_HR_, and needle track (orange line) for: (a) needle inserted into CTV_HR_ and (b) needle inserted into the uterus.

Current limitations of the phantom include its single‐use due to needle tracks in the agar after insertion, variability in silicone quality, and limitation to a single representative anatomical model. The one‐time use limitation can be mitigated by replacing the agar, allowing for the silicone anatomy to be reused. Silicone quality can be improved using vacuum degassing to remove bubbles that lead to US signal degradation. The current representative model includes an idealized probe position with a rectal length that allows full visibility of the uterine fundus. In clinical practice, there may be cases where the rectum position does not allow TRUS visibility of deep‐seated targets. For those cases, a hybrid TRUS/TAUS approach may need to be considered.^22^

Future work with this phantom will include incorporation of the tracking system developed by Eigen Health Services LLC to image, guide and track needles into a target volume with the goal of improving insertion position accuracy and efficiency. We hypothesize that improved accuracy via real‐time tracking will reduce overall implant times by reducing the number of post‐insertion images and needle repositioning. It will also aid in reducing tissue trauma and risk of infection and injury, resulting in fewer overall needles placed in order to achieve an optimal implant.

## 5 CONCLUSION

A novel low‐cost, multi‐modality, anatomically realistic female pelvic phantom using a mold‐based design for intracavitary/interstitial HDR brachytherapy applications was developed. An agar formulation and Smooth‐On Mold Star™ Silicone 20T (Smooth‐On, Easton, PA) were implemented for similarities in mechanical properties to the female pelvic anatomy. This multi‐modality phantom will be used to validate the feasibility of introducing an intraoperative imaging technique using TRUS applications into clinic workflow to aid in more accurate HDR needle placement.

## AUTHOR CONTRIBUTIONS

Brett Eckroate: lead author, phantom design and construction, data acquisition, manuscript revision, and final review. Julie Raffi and Oana Craciunescu: conception and design of study, data acquisition, manuscript revision, equal contributions as senior authors. Diandra Ayala‐Peacock: conception and design of study, manuscript review. Rajesh Venkataraman: conception and design of study, equipment vendor. Sabrina Campelo: data acquisition, manuscript review. Junzo Chino, Sarah Jo Stephens, Yongbok Kim, and Sheridan Meltsner: data review, manuscript review.

## CONFLICT OF INTEREST STATEMENT

The authors declare no conflicts of interest.
